# Transmural "Scar-to-Scar" Reentrant Ventricular Tachycardia

**DOI:** 10.1016/s0972-6292(16)30690-8

**Published:** 2013-11-15

**Authors:** Jason S Bradfield, Roderick Tung, Kalyanam Shivkumar

**Affiliations:** UCLA Cardiac Arrhythmia Center, David Geffen School of Medicine at UCLA, Los Angeles, CA

**Keywords:** Reentrant Ventricular Tachycardia, Transmural Scar

## Abstract

We describe a scar-related reentrant ventricular tachycardia circuit with a proximal segment in an endocardial basal septal scar and an exit in a region of slow conduction in a non-overlapping region of epicardial basal lateral scar. The 12-lead EKG demonstrates criteria for a basal lateral epicardial VT, however the same morphology could be produced with a longer stim-latency with pace mapping or VT induction from the endocardial septal region of scar. A significant segment of myocardium demonstrated no endocardial or epicardial scar on electroanatomic mapping, suggesting the presence of a mid-myocardial isthmus. Further evidence was provided by assessment of unipolar settings. The epicardial VT that initially appeared to originate from the basal lateral epicardial region, was successfully treated with radiofrequency ablation of the lateral aspect of the endocardial septal scar.

## Case

We present a 46 year-old man with a history of non-ischemic cardiomyopathy (CM), likely of familial origin, status-post implantable cardioverter-defibrillator (ICD) implantation who was transferred to our center after presenting with ventricular tachycardia (VT) storm and recurrent ICD shocks for monomorphic VT, with a rate between 165-175 beats-per-minute, despite therapy with antiarrhythmic medications.

**Figure 1:**
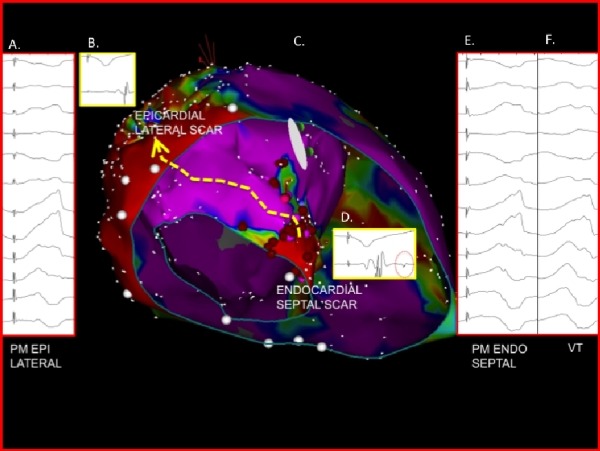
Epicardial voltage map superimposed on the endocardial voltage map demonstrating the circuit of activation for the induced VT. A) Epicardial pacemap with stim-latency of 30 ms; B) Delayed, but not late potential on epicardial surface; C) Superimposed endocardial and epicardial voltage maps; D) Endocardial late potential; E) Endocardial pace map with stim-latency of 115 ms; F) 12-lead of induced ventricular tachycardia with QRS duration of 192 ms.

The patient underwent endocardial and epicardial mapping as is the protocol at our institution for any patient with a non-ischemic cause of their CM. Endocardial and epicardial electroanatomic maps (EAM) were obtained with a Carto 3 system (Biosense Webster, Diamond Bar, CA). (Figure 1c)

Monomorphic VT was induced with double extrastimuli testing. Standard 12-lead EKG morphology demonstrated a VT with a right bundle branch block pattern, V5 transition and inferior axis at a rate of 170 beats-per-minute (Figure 1f). The EKG characteristics met criteria for an epicardial exit based on a pseudo-delta wave >75ms and an MDI >0.59. [1] The VT was not hemodynamically tolerated and therefore a substrate based approach targeting late potentials and utilizing pace mapping was required.

An excellent pace-map could be produced from the basal lateral epicardial surface (Figure 1a) with a short stim-QRS latency of 30 ms, which was 16% of the tachycardia cycle length (TCL) consistent with an exit site. No late potentials (Figure 1b) were seen on the epicardial surface and therefore we proceeded with trans-septal catheterization and voltage mapping of the left ventricular endocardium.

**Figure 2:**
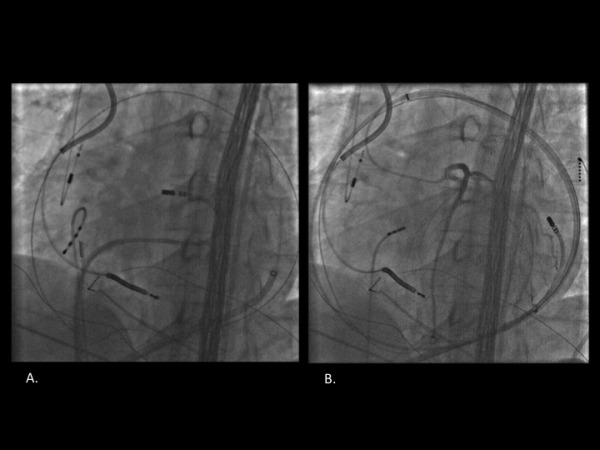
LAO fluoroscopic location of endocardial/proximal pace-map site (A) and radiographically distant epicardial/exit pace-map site (B).

**Figure 3:**
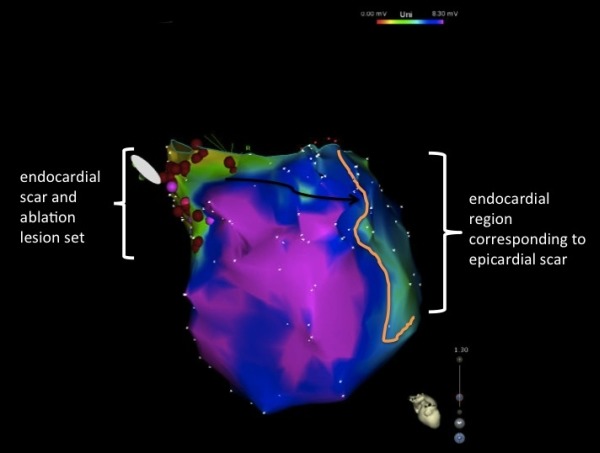
Endocardial unipolar voltage map with setting of 8.3 mV. Unipolar setting demonstrates evidence of intramural scar which is the potential substrate to link the endocardial septal scar and the epicardial lateral scar to form a circuit for reentry. Orange line = border of corresponding epicardial scar region. Black arrow = region of myocardium with no endocardial or epicardial scar that the proposed circuit must travel.

Interestingly, at a late potential site (Figure 1d) within the endocardial basal septal scar, a similarly well matched pace-map of the tachycardia was seen with a longer stim-QRS latency of 115 ms, which was 60% of the TCL consistent with a proximal isthmus site. (Figure 1e) Additionally, as recently described by our group, a pace map induction (PMI) and multiple exits sites (MES) were seen from a region of slow conduction within the endocardial basal septal scar. [2] There was no endocardial basal lateral scar to correspond to the epicardial basal lateral scar and further there was a larger region of the anterior wall between the two regions of scar that had no discernable scar on the endocardial or epicardial surfaces. (Figure 2 and 3) Assessment of unipolar setting based on the work of Hutchinson and colleagues [3] suggested the presence of a region of abnormal voltage that may provide an intramural connection between the endocardial and epicardial scars. (Figure 3)

Radiofrequency ablation of the lateral aspect of the septal endocardial scar at sites of late and fractionated potentials was undertaken. After ablation of this region the clinical tachycardia was non-inducible. The patient was free of VT at greater than 9 months follow-up off of antiarrhythmic medications.

## Discussion

The above findings demonstrate that the VT exit site was the basal lateral epicardium. The findings of no corresponding endocardial basal lateral scar with endocardial septal pace-maps that were matches to the epicardial pace-maps at a lateral site, in combination with a VT that could be induced from the endocardial septal region with a PMI suggests a mid-myocardial connection between the two scar regions that was not adequately visualized with standard EAM system settings.

This is the first description in the literature we are aware of demonstrating a VT circuit that likely required a bridging mid-myocardial segment for non-overlapping regions of scar on the endocardium and epicardium. While current practice and available mapping techniques lead electrophysiologists to label VT as having an endocardial or epicardial origin, myocardial scar formation does not follow an either/or pattern. Rather many, if not most, myocardial scars that predispose to reentry likely involve a complex three-dimensional substrate that may include the endocardium, mid-myocadium and epicardium. This finding has been reported in a canine model [4]. However, there is little data regarding this concept in humans but the limited available data suggests that mid-myocardial circuits may be even more common in a non-ischemic CM population.

Anter and colleages [5] reported their experience with surgical ablation after failed percutaneous ablation. They report that at least 4/8 patients in their series had mid-myocardial scar seen on cMRI and that when detailed electroanatomic maps were obtained there was no corresponding scar seen at the corresponding endocardial or epicardial sites. Further work has demonstrated that cardiac MRI and EAM systems with appropriate settings can demonstrate extension of scar vital to arrhythmia origin that are distant from the endocardial surface, including intramural scar, and that unipolar voltage settings may be of particular use in this setting. However, much of this work was done in ischemic CM, but data on the non-ischemic CM patient population is beginning to emerge and needs further study.

These early studies and this case raises the importance of a complete electroanatomic map with pacemapping used as an adjunct technique for scar-related reentry, especially in a situation where the initially proposed exit site of the tachycardia demonstrates no late potential activity in a hemodynamically untolerated VT.

Lastly, this case illustrates that 12-lead morphology is only predictive of the exit site of a reentrant circuit. There are likely many VT morphologies that indicate the need for epicardial mapping and ablation with subendocardial or intramural isthmuses and vice versa.

The use of pace-mapping and not entrainment mapping is a limitation of this manuscript. Stim-QRS latency during pacing is not necessarily synonymous with Stim-QRS during entrainment. However, the authors interpret the longer stim-QRS latency to be similarly indicative of progressive distance from the exit site and evidence that in this case there was a single preferential exit site toward the lateral epicardium.
